# An algorithm for determining scoliosis curve type according to Schroth

**DOI:** 10.1186/1748-7161-7-S1-O53

**Published:** 2012-01-27

**Authors:** S Schreiber, EC Parent, EM Watkins, DM Hedden

**Affiliations:** 1 University of Alberta, Faculty of Rehabilitation Medicine Edmonton, Canada; 2 University of Alberta/Alberta Health Services, Edmonton, Canada

## Objective

To describe a refined classification algorithm, based on instructions provided during certification training, to unambiguously guide scoliosis curve type classification in a clinical trial.

## Background

Schroth exercises are scoliosis specific [1,2]. They are the most researched and have been shown to lead to good outcomes. The Schroth classification consists of four mutually exclusive curve type categories (3c, 3cp, 4c and 4cp). Patients with scoliosis are classified according to their clinical presentation by a certified Schroth therapist. Observing the alignment of the following body blocks guides the classification assessment: lumbar spine and pelvis, thoracic spine and rib cage, and the cervical spine, head and shoulder girdle. Classifying patients’ curve types within the four Schroth curve categories determines the appropriate exercise prescription for a patient. An algorithm is needed to minimize errors in classifying different scoliosis patterns and help standardize exercise prescription.

## Materials and methods

Using the Schroth classification instructions described by Hennes, A. (2008, 2009), two certified Schroth research therapists and a physiotherapy professor developed the proposed algorithm and a set of operational definitions and instructions with respect to:

• major curve location

• body blocks rotation

• relative position of the pelvis and lumbar block

• prominent hip location

• body weight balance

## Results

A refined algorithm for determining the scoliosis curve type according to Schroth is proposed for use in a randomized clinical trial.

## Conclusions

Using the proposed algorithm may help prevent treatment failure by minimizing incorrect classification and exercise prescription. The algorithm can help clinicians correctly classify curves, which then allows an appropriate exercise prescription.
